# Effects of energy and amino acid intake during gestation on reproductive performance, milk composition, antioxidant status and placental nutrient transport in high-parity sows

**DOI:** 10.3389/fvets.2025.1585925

**Published:** 2025-04-28

**Authors:** Liang Hu, Jie Zheng, Fali Wu, Zhengfeng Fang, Lianqiang Che, De Wu

**Affiliations:** ^1^Key Laboratory for Animal Disease-Resistance Nutrition of China Ministry of Education, Institute of Animal Nutrition, Sichuan Agricultural University, Chengdu, Sichuan, China; ^2^Key Laboratory of Agricultural Product Processing and Nutrition Health (Co-construction by Ministry and Province), Ministry of Agriculture and Rural Affairs, College of Food Science, Sichuan Agricultural University, Ya'an, China

**Keywords:** energy, amino acids, fetal survival, antioxidant, high-parity sows

## Abstract

Appropriate nutritional strategies show promise for enhancing productive performance and longevity of sows. This study aimed to determine the effects of increased energy and amino acids (AA) intake during gestation on reproductive performance, milk composition, blood parameters, placental nutrient transport and antioxidant capacity of high-parity sows. A total of 72 Landrace × Yorkshire sows (seventh to ninth parity) were randomly assigned to dietary treatments consisting of combinations of 2 energy levels and 2 AA levels in a 2 × 2 factorial design. Blood and milk samples and placentas from sows were collected to measure biochemistry parameters, milk composition, antioxidant indexes, and indicators related to nutrient transport. The results showed that sows fed high energy reduced the number of live born piglets (*p* < 0.05), increased the birth weight of piglets (*p* < 0.05). Further observation showed that sows fed high energy decreased plasma progesterone at 30 and 60 days of gestation and plasma glutathione peroxidase (GPX) and total superoxide dismutase (T-SOD) at 90 days of gestation (*p* < 0.05), increased fat content in colostrum and average daily weight gain of piglets during lactation (*p* < 0.05). Moreover, high energy intake during gestation decreased mRNA expression of GPX and increased mRNA expression of glucose transporter 3 in the placenta (*p* < 0.05). High AA intake during gestation showed a tendency to increase litter birth weight and colostrum protein content (*p* < 0.10), and increased plasma urea nitrogen at day 110 of gestation and plasma T-SOD at day 90 of gestation (*p* < 0.05). Furthermore, sows fed high AA intake during gestation increased mRNA expressions of placental extracellular SOD and sodium-dependent neutral amino acid transporter 1 (*p* < 0.05). In conclusion, sows feed high energy during gestation negatively impacted fetal survival by reducing progesterone concentrations and antioxidant capacity, while simultaneously increasing piglet birth weight through enhanced placental nutrient transport. Moreover, sows received high AA during gestation exhibited better redox status and improved litter performance, which can be attributed to enhancements in antioxidant capacity and colostrum composition.

## 1 Introduction

Sow longevity is critical to the overall success and profitability of commercial swine operations. Consequently, a sow should not be culled from the breeding herd as long as her reproductive performance remains within an acceptable range (1). For animal nutritionists, it is essential to enhance nutritional strategies to extend longevity by maximizing productivity in high-parity sows.

Modern prolific sows require improved nutrient intake and feeding strategies due to their larger body size and the substantial decline in body mass during lactation (2, 3). Achieving optimal reproductive output depends on the sow consuming adequate amounts of energy and nutrients throughout gestation (4, 5). Previous studies have underscored the importance of appropriate nutrition during gestation for fetal development and growth, which ultimately affects piglet survivability and litter performance (6, 7). Furthermore, optimizing nutrient intake during gestation can enhance the longevity of sows by preventing excessive body weight loss during lactation and shortening the weaning-to-estrus interval (5, 8).

Daily requirements of energy and AA can be calculated factorially throughout the entire gestation period (7, 9). The recommended daily nutrient requirements for multiparous sows are slightly lower than those for primiparous sows, due to the mature body condition which reduces the nutrient requirement for growth (2, 10). Specifically, current guidelines indicate a lower standardized ileal digestible (SID) Lys level for sows of fourth parity and above compared to younger sows (9). Emerging evidence indicates that increasing nutrient intake during late gestation may enhance reproductive performance in multiparous sows (6, 10). However, it remains unknown whether these lower recommendations adequately meet the nutritional needs of high-parity sows, particularly in relation to embryo survival, fetus growth, and colostrum synthesis.

The placenta serves as a vital interface for fetal nourishment. It has well demonstrated that enhancing maternal nutrient intake can promote fetal growth by improving placental transport efficiency (11). Furthermore, fetal development and growth processes are highly susceptible to maternal oxidative stress (12). We have previously established high energy and AA intake during late gestation altered the redox status of sows at farrowing (6). Nonetheless, it is unclear whether increased energy or/and AA intake throughout gestation affect the antioxidant ability of the placenta in high-parity sows. We hypothesized that optimized energy and AA intake during gestation may lead to better reproductive performance and litter growth through altering plasma metabolites, placental nutrient transporter and antioxidant ability. To test this hypothesis, we conducted a factorial design experiment to evaluate the effects of energy and AA intake during gestation on reproductive performance, blood metabolites, and gene expression related to nutrient transporter and oxidative stress in the placenta of high-parity sows. This investigation may provide mechanistic insights into nutritional programming strategies for high-parity sows.

## 2 Materials and methods

### 2.1 Ethics approval

The experiment followed the actual law of animal protection and was approved by the Animal Care and Use Committee of the Sichuan Agricultural University (Ethic Approval Code: SCAUAC201408-3), and was performed in accordance with the National Research Council’s Guide for the Care and Use of Laboratory Animals.

### 2.2 Animals, diets and experiment design

Seventy-two Landrace × Yorkshire sows (parity 7–9) with initial body weight (BW, 235.08 ± 4.7 kg) and backfat thickness (18.72 ± 0.7 mm) were used in this study. After artificial insemination, sows were randomly divided into to 4 dietary treatments (*n* = 18 sows/group), which consisted of the combinations of 2 total AA levels [0.69 or 0.40% Lys, other AA were calculated according to their ratio to Lys indicated by NRC (2012), all AA levels met or exceeded the NRC (2012) recommendations] and 2 energy levels [3.0 or 3.4 Mcal/kg (Digestible energy, DE)] in a 2 × 2 factorial design; the details are shown in Table 1. This produced four experimental groups (Energy-AA): Low energy-Low AA (LE-LAA), Low energy-High AA (LE-HAA), High energy-Low AA (HE-LAA) and High energy-high AA (HE-HAA). All sows were received the same lactation diet.

**Table 1:** Ingredient and nutrient composition of diets for the pregnant sows.

Item	Low energy		High energy	
	High AA	Low AA	High AA	Low AA
Ingredients, %				
Corn	62.12	47.00	60.84	47.00
Soybean meal (43%)	16.67	–	18.75	–
Soybean oil	–	–	5.70	7.80
Wheat bran (98%)	13.20	22.40	8.35	22.92
Rice bran and hull	3.40	2.19	–	–
Cellulose	0.63	1.76	2.35	2.69
Corn starch	–	22.28	–	15.20
_L_-lysine HCl (98.5%)	0.02	0.20	–	0.20
_DL_-methionine (99%)	0.04	0.03	0.05	0.03
_L_-threonine (98.5%)	0.08	0.10	0.07	0.10
_L_-tryptophan (98%)	–	0.02	–	0.02
Choline chloride (50%)	0.15	0.15	0.15	0.15
Limestone	1.55	1.61	1.55	1.60
Dicalcium phosphate	1.10	1.22	1.15	1.25
Salt	0.50	0.50	0.50	0.50
Vitamin and mineral premix^1^	0.54	0.54	0.54	0.54
Total	100.00	100.00	100.00	100.00
Nutrient content				
Digestible energy, Mcal/kg	3.00	3.00	3.40	3.40
Crude protein, %	14.10	7.30	14.10	7.30
Lysine, %	0.69	0.40	0.69	0.40
Methionine + cystine, %	0.51	0.29	0.51	0.29
Threonine, %	0.61	0.34	0.61	0.34
Tryptophan, %	0.16	0.09	0.16	0.09
Calcium, %	0.90	0.90	0.90	0.90
Available phosphorus, %	0.31	0.31	0.31	0.31
Crude fiber, %	5.00	5.00	5.00	5.00

^1The premix supplied the following vitamins and trace minerals per kilogram of diet: vitamin A, 0.94 mg; vitamin D3, 0.01 mg; vitamin E, 20 mg; vitamin K3, 1 mg; vitamin B12, 0.04 mg; riboflavin, 5 mg; niacin, 20 mg; pantothenic acid, 15 mg; folic acid, 1.5 mg; thiamin, 1.5 mg; pyridoxine, 2 mg; biotin, 0.1 mg; Cu 6.0 mg; Zn, 90 mg; Mn, 40 mg; Fe, 90 mg; Se, 0.3 mg; I, 0.2 mg.^

### 2.3 Feeding managements

During gestation, all sows received one of the four diets twice daily (at 0800 and 1,500 h, half of the daily meal each time). They were placed on a three-stage feeding program during gestation as follows: phase 1 spanned from day 1 of gestation (G1) to G30 (2.2 kg/day), phase 2 ranged from G31 to G90 (2.4 kg/day), and phase 3 ranged from G91 to parturition (2.8 kg/day). After farrowing, sows were offered the lactation diet three times daily (at 0800, 1200, and 1,500 h), starting at 2.0 kg/day, then gradually increasing by 1 kg/day until the sixth day, after which the sows feed ad libitum. Throughout the experiment, sows had free access to water. Sows were individually housed in pens measuring 2.6 m × 0.6 m until G107, then moved to the farrowing room and placed in individual farrowing crates measuring 2.2 m × 1.5 m. All sows were housed in the same barn and subjected to controlled environmental conditions, the ambient temperature was maintained at 20–25°C, with humidity range from 40 to 60%. Heating light and pads were provided for suckling piglets, maintaining the temperature between 26°C and 32°C, which was gradually decreased with increase in age.

### 2.4 Sampling and data collection

The sow BW was measured in the morning (0800 h) at G1, G110 and day 28 of lactation, while sow BW after farrowing was estimated by deduction of total litter weights of piglets and placenta from BW at G110. Meanwhile, backfat thickness was measured ultrasonically (Renco Lean-Meater, United States) at the level of the last rib on each side, 65 mm from the midline. Total number of piglets born, including the number of piglets born alive, number of stillborn, and number of mummified fetuses, were recorded as described previously (13). All piglets were weighed at birth, cross-fostered within the same group beyond 24 h and weighed each week for calculating piglet average daily weight gain (ADG) and litter weight at weaning. The average daily feed intake (ADFI) of the sows during lactation was recorded.

Sow fasting blood samples were collected from the jugular vein before the morning meal on day 0, 30, 60, 90, 110 of gestation and on day 28 of lactation. Samples were collected in heparinized tubes kept on ice and centrifuged at 2,550 × *g* for 10 min at 4°C, then stored at −80°C until analysis. Colostrum samples were collected from each sow within 1 h after onset of farrowing as described previously (6). Milk samples were obtained from all functional glands at day 14 and 28 of lactation after 0.3 mL oxytocin (Ocytovem, CEVA, Santé Animale, Libourne, France) was injected into the ear vein of each sow. Colostrum and milk samples were immediately filtered through gauze, aliquoted and stored at −20°C immediately prior to subsequent analysis. After parturition, six piglets (one male piglet from each group of six litters), whose BW was closest to the average BW of their litter, were selected and anesthetized with an intravenous injection of Zoletil 50^®^ (0.1 mg/kg BW) before jugular exsanguination as previously reported (14). After the abdomen was exposed, the heart, lung, spleen, pancreas, kidney, stomach and liver were quickly removed and weighed. Samples of chorioallantois were obtained from the placental of piglets with a BW closest to the average BW as described previously (15). All the samples were snap-frozen in liquid nitrogen and then stored at fridge with −80°C until further analysis.

### 2.5 Determination of colostrum and milk composition

To evaluate the composition of colostrum and milk, frozen samples (*n* = 8) were thawed at 4°C for composition analysis. The fat, protein, lactose, solids-non-fat and ash content were measured using a milk composition analyzer (Milkoscan 4,000; Foss MilkoScan, Hillerød, Denmark) as described previously (3).

### 2.6 Measurement of plasma antioxidant parameters

Plasma samples (*n* = 8) were used to measure malondialdehyde (MDA; catalog no. A003-1-2), total superoxide dismutase (T-SOD; catalog no. A001-1-2) and glutathione peroxidase (GPX; catalog no. A005-1-2) using specific assay kits (Nanjing Jiancheng Bioengineering Institute, Nanjing, China). The plasma concentration of MDA was measured using thiobarbituric acid method as described previously (16). The sample was incubated with MDA standard in water bath at 95°C and reacted with thiobarbituric acid, and then the sample and MDA standards were extracted through alcohol and finally the sample and MDA standards were measured for absorbance at 532 nm spectrophotometrically. The concentration of plasma T-SOD was measured according to the method as described previously (17). T-SOD has specifically ability to inhibit the free radicals of superoxide anion, thereby reducing the production of nitrite, and nitrite can be measured spectrophotometrically. The absorbance was measured at 550 nm and the concentration of plasma T-SOD was calculated by reduced nitrite. The concentration of plasma GPX was determined according to the method as described previously (18). The activity of GPX was expressed by the rate of oxidation of GSH, and the absorbance of the plasma sample and the standard were measured at 412 nm spectrophotometrically. All of plasma samples were measured in duplicate and the mean values were used for statistical analysis.

### 2.7 Measurement of plasma biochemical parameters

Plasma concentrations of urea N, glucose, triglyceride and cholesterol (*n* = 8) were measured by automatic biochemical analyzer (Model 7,020, Hitachi, Tokyo, Japan) according to corresponding commercial kits (Sichuan Maker Biotechnology Inc., Chengdu, China). There was less than 5% variation of intra- and inter-assay coefficients for each assay.

### 2.8 Determination of plasma progesterone

Plasma levels of progesterone (*n* = 8) were determined using a specific enzyme-linked immunosorbent assay kit (TSZ, Shanghai, China). The assays were performed following the manufacturer’s instructions. The absorbance (optical density value) of the samples was measured at 450 nm using a microplate reader, and the measurement was conducted within 15 min of terminating the reaction. The intra- and inter-assay coefficients of variation were 11 and 15%, respectively.

### 2.9 Real-time quantitative reverse transcriptase PCR

Total RNA was extracted from approximately 100 mg of frozen placental tissues (*n* = 8) using Trizol Reagent (Invitrogen, Carlsbad, CA, United States) according to the manufacturer’s instructions. RNA integrity and quality were assessed via agarose gel electrophoresis (1%) and spectrophotometric analysis (A260/A280 ratio). RNA concentration was determined using a nucleic-acid/protein analyzer (Beckman DU-800, CA, United States). Following RNA concentration determination, 1 μg of total RNA was reverse-transcribed into complementary DNA (cDNA) using the PrimeScripte RT Reagent Kit (TaKaRa Biotechnology, Dalian, Liaoning, China). The resultant cDNA was stored at −20°C for subsequent relative quantification by polymerase chain reaction (PCR). Primers were designed using Primer Express 3.0 (Applied Biosystems, Foster City, CA, United States) and listed in Table 2. cDNA amplification was performed using an ABI 7900HT real-time PCR system (Applied Biosystems, United States). The mixture (10 μL) contained 5 μL of SYBR Green Supermix (TaKaRa, Japan), 1 μL of cDNA, 0.4 μL of each primer (10 μM), 0.2 μL of ROX Reference Dye and 3 μL of ddH_2_O. The cycling conditions were used as follows: denaturation at 95°C for 15 s, followed by 40 cycles of denaturation at 95°C for 5 s, annealing at 60°C for 30 s, and extension step at 72°C for 15 s. Product size was determined by agarose gel electrophoresis. The standard curve of each gene was run in duplicate and three times for obtaining reliable amplification efficiency values as described previously (17). The correlation coefficients (r) of all the standard curves were >0.99 and amplification efficiency values were between 90 and 110%. The most stable housekeeping genes (*β-actin* and *GADPH*) were chosen for normalization. Relative mRNA abundance was determined using the *Δ* cycle threshold (ΔCt) method, as outlined in the protocol of Applied Biosystems. Briefly, a ΔCt value is the Ct difference between the target gene and the reference gene (ΔCt = Ct^target^ – Ct^reference^). For each of the target genes, the ΔΔCt values of all the samples were calculated by subtracting the average ΔCt value of the corresponding LE-HAA group. The ΔΔCt values were converted to fold differences by raising 2 to the power –ΔΔCt (2^–ΔΔCt^) according to previous study (19).

**Table 2:** Primer sequences of target and reference genes.

Gene	Genebank accession no.	Primer sequence	Amplification length (bp)
*CAT*	NM_214301.2	5′-ACTTCTGGAGCCTACGTCCT-3′	93
		5’-ATCCGTTCATGTGCCTGTGT-3′	
*iNOS*	NM_001143690.1	5’-AGAGCCTCTGGACCTCAACA-3′	136
		5’-CTCACAGCAGAGTTCCACCA-3′	
*GP_X_*	NM_214201.1	5’-GCTCGGTGTATGCCTTCTCT-3′	103
		5’-AGCGACGCTACGTTCTCAAT-3′	
*ESOD*	NM_001078688.1	5’-ACGCTGCTCTGTGCTTACCT-3′	142
		5’-TCAACTCCTGCCAGATCTCC-3′	
*Nrf2*	XM_003133500.5	5’-GCCCCTGGAAGCGTTAAAC-3’	67
		5’-GGACTGTATCCCCAGAAGGTTGT-3’	
*Keap-1*	NM_001114671.1	5’-ACGACGTGGAGACAGAAACGT-3’	56
		5’-GCTTCGCCGATGCTTCA-3’	
*SNAT1*	XM_003355629.4	5’-TTTTCTTGGGTCTGGGGGTG-3’	80
		5’-ACTACTTGATGAGCAGGCCC-3’	
*SNAT2*	NM_001317081.1	5’-AGCCGTAGAAGAATGATGAATGTGTCC-3’	128
		5’-TAGGTGTGAAGCAATTCCGTCTCAAC-3’	
*SNAT4*	XM_021092582.1	5’-GCCAACACAGGGATCATACT-3’	100
		5’-CTCCTTCCTTGGAGGTCTTTAAT-3’	
*FATP1*	XM_021076151.1	5’-GCCAGATCGGCGAGTTCTAC-3’	71
		5’-ACCAACCTTCCCATCCATGTT-3’	
*GLUT1*	XM_005665507.1	5’-TCCTTCAGCCAGCAGTGATG-3’	180
		5’-AGCGTGGGATGTGGGTAAAG-3’	
*GLUT3*	XM_003355585.3	5’-GCCTTGACCTTTCCCATAGACA-3’	111
		5’-CTACTTCCACCCAGAGCAAAGT-3’	
*β-actin*	DQ845171.1	5’-GGCGCCCAGCACGAT-3’	66
		5’-CCGATCCACACGGAGTACTTG-3’	
*GAPDH*	NM_001206359.1	5’-TCGGAGTGAACGGATTTGGC-3’	147
		5’-TGCCGTGGGTGGAATCATAC-3’	

CAT, catalase; iNOS, nitric oxide synthase 2, inducible; GPX, glutathione peroxidase; ESOD, extracellular superoxide dismutase; Nrf2, nuclear erythroid 2-related factor 2; Keap1, Kelch-like ECH-associated protein 1; GAPDH, glyceraldehyde-3-phosphate dehydrogenase; GLUT, glucose transporter 1; FATP, fatty acids transport protein; SNAT, sodium-dependent neutral amino acid transporter.

### 2.10 Calculations and statistical analysis

The protein and fat mass of sows were estimated from body weight and backfat thickness measurements using the equations according to previous study (20), as follows:

1. EBW = 0.905 × BW^1.1013^;
2. Lipids (kg) = −26.4 + 0.221 × EBW + 1.331 × P_2_;
3. Protein (kg) = 2.28 + 0.178 × EBW −0.333 × P_2_,

where EBW (kg) represents the sow empty live weight, BW (kg) represents the sow live weight, and P_2_ (mm) represents backfat thickness at the last rib. The individual sow was regarded as the experimental unit and piglet data was reported as a mean for the litter. All data were checked for outliers and normality before being analyzed. Results are presented as means with their standard errors. Data were analyzed as repeated measures using the MIXED procedure of Statistical Product and Service Solutions 20.0 (Chicago, IL, United States) according to the following model: *y_ijk_* = *μ* + *a_i_* + *b_j_* + (*ab*)*_ij_* + *e_ijk_* (*_i_* = 1, 2, *_j_* = 1, 2, *_k_* = 1, 2,…), where *y_ijk_* represents the dependent variable, *μ* is the mean, *a_i_* is the effect of Energy (High, Low), *b_j_* is the effect of amino acid (High, Low), (*ab*)*_ij_* is the interaction between Energy and AA, and *e_ijk_* the error term. Duncan’s multiple range test was used for multiple comparison *post hoc* when the effects of BW, Diet or their interaction was significant. To assess the correlation between reproductive performance and blood parameters and genes expressions of sows, Pearson’s correlation test was performed using GraphPad Prism 7.00 for Windows (GraphPad Software, San Diego, CA, United States), and results are represented by Pearson correlations (*r*) and level of significance (*P*). Differences were considered as significant when *p* < 0.05, and a tendency was recognized when *p* < 0.10.

## 3 Results

### 3.1 Body condition and reproductive performance

Sows had a greater body weight gain when fed high energy (*p* < 0.001) or high AA (*p* < 0.001) during gestation, the same results were observed in back fat gain of sows fed high energy (*p* = 0.009) or high AA (*p* = 0.068; Table 3). Furthermore, body weight gain and back fat gain were greatest in sows fed HE-HAA diet and lowest in sows fed LE-LAA diet (Table 3). Sows fed high energy had a lower number of born live piglets (*p* = 0.016) and tended to increase the number of mummified fetuses (*p* = 0.060). However, the average birth weight of piglet was higher in sows with high energy intake (*p* = 0.001). In addition, sows with high AA intake tended to improve the average birth weight of piglet (*p* = 0.094; Table 3).

**Table 3:** Effects of energy and amino acids intake during gestation on body condition and reproductive performance of sows.

Item	LE		HE		SEM	*p*-value		
	HAA	LAA	HAA	LAA		Energy	AA	Energy × AA
Backfat, mm								
Breeding	18.84	18.70	18.78	18.56	0.33	0.886	0.790	0.952
d 110 of gestation	20.91	20.45	21.70	20.84	0.34	0.400	0.351	0.780
Back fat gain	2.07^a^	1.75^a^	2.91^b^	2.29^a,b^	0.14	0.009	0.068	0.544
Body weight, kg								
Breeding	236.21	232.34	235.87	235.89	3.08	0.801	0.762	0.759
d 110 of gestation	283.02	268.29	287.25	281.69	3.30	0.181	0.125	0.483
Body weight gain	46.81^b,c^	35.94^a^	51.38^c^	45.80^b^	1.15	<0.001	<0.001	0.150
Total born piglets, no.	13.00	13.07	12.06	12.00	0.58	0.211	0.998	0.928
Live born piglets, no.	11.46	11.14	9.79	9.66	0.34	0.016	0.708	0.900
Stillborn, no.	0.87	1.00	0.86	0.81	0.18	0.738	0.881	0.763
Mummified fetuses, no.	0.67	0.93	1.43	1.56	0.18	0.092	0.628	0.875
Live born, %	88.93	85.86	83.86	81.75	0.02	0.208	0.475	0.894
Stillborn, %	6.40	7.29	5.43	6.13	0.01	0.607	0.702	0.964
Mummified fetuses, %	4.61	6.71	10.71	12.19	0.01	0.060	0.555	0.916
Total born								
Litter birth weight, kg	15.90	14.01	14.71	14.41	0.28	0.619	0.168	0.313
Piglet birth weight, kg	1.28^a,b^	1.13^a^	1.44^b^	1.42^b^	0.05	0.001	0.107	0.259
Birth weight CV, %	20.34	21.38	19.72	18.51	0.80	0.314	0.962	0.514
Live born								
Litter birth weight, kg	15.44	13.35	14.07	13.77	0.36	0.600	0.094	0.196
Piglet birth weight, kg	1.31^a,b^	1.17^a^	1.45^b^	1.43^b^	0.03	0.001	0.114	0.260
Birth weight CV, %	20.21	20.97	19.54	18.43	0.72	0.344	0.673	0.436

Sows were regarded as the experimental units, *n* = 18 for each group. Mean values with their standard error. Mean values within a row with unlike superscript letters were significantly different (*p* < 0.05). LE, low energy intake; HE, high energy intake; HAA, high amino acids intake; LAA, low amino acids intake.

### 3.2 Organ indices of newborn piglets

As shown in Table 4, piglets from sows fed high energy tended to increase the relative weight of liver to BW (*p* = 0.094), but had a lower relative weight of brain to BW (*p* < 0.001; Table 4). Moreover, piglets from sows fed high AA tended to increase the relative weight of kidney to BW (*p* = 0.092).

**Table 4:** Effect of energy and AA intake during gestation on the organ indices of newborn piglets.

Item	LE		HE		SEM	*P*-value		
	HAA	LAA	HAA	LAA		Energy	AA	Energy × AA
Birth weight, kg	1.29^b^	1.06^a^	1.40^b^	1.41^b^	0.03	<0.001	0.013	0.012
Body L, cm	35.48	35.52	36.32	37.13	0.58	0.333	0.734	0.759
Head L, cm	10.04	9.72	10.61	10.60	0.16	0.032	0.605	0.629
BMI, kg/m^2^	10.31	8.44	10.90	10.42	0.41	0.124	0.157	0.395
Relative weight, %								
Heart wt: BW	0.75	0.73	0.75	0.78	0.03	0.695	0.983	0.630
Liver wt: BW	2.22	2.14	2.53	2.65	0.11	0.094	0.915	0.662
Spleen wt: BW	0.08	0.07	0.08	0.08	0.00	0.934	0.337	0.189
Lung wt: BW	1.53	1.52	1.37	1.55	0.07	0.675	0.564	0.493
Kidney wt: BW	0.86^b^	0.67^a^	0.81^a,b^	0.80^a,b^	0.03	0.531	0.092	0.135
Stomach wt: BW	0.49	0.48	0.48	0.45	0.01	0.483	0.441	0.730
Pancreas wt: BW	0.11	0.09	0.09	0.10	0.01	0.502	0.834	0.224
Brain wt: BW	2.12^b^	2.39^b^	1.75^a^	1.78^a^	0.07	<0.001	0.156	0.247

Sows were regarded as the experimental units, *n* = 6 for each group. Mean values with their standard error. Mean values within a row with unlike superscript letters were significantly different (*P* < 0.05). LE, low energy intake; HE, high energy intake; HAA, high amino acids intake; LAA, low amino acids intake; L, length; BMI, body mass index; BW, body weight; wt, weight.

### 3.3 Litter performance during lactation

Sows fed high energy had a smaller litter size after cross-foster and at weaning (*p* < 0.05; Table 5). The average BW of piglets were higher in sows fed high energy (*p* = 0.003), whereas sows fed high AA tended to increase the average BW of piglets (*p* = 0.079) at weaning. Sows fed both high energy (*p* = 0.058) and high AA (*p* = 0.078) increased the litter weight during the first 2 weeks. The average ADG of piglets in the entire lactation was higher in sows fed high energy than that in sows fed low energy (*p* = 0.018). In addition, sows fed high energy tended to decrease the average feed intake during lactation (*p* = 0.058).

**Table 5:** Effect of energy and amino acids intake during gestation on litter performance and feed intake of sows during lactation.

Item	LE		HE		SEM	*p*-value		
	HAA	LAA	HAA	LAA		Energy	AA	Energy × AA
Litter size. No/litter								
After cross-foster	10.67	10.64	9.79	9.63	0.18	0.009	0.795	0.847
At weaning	10.47	10.43	9.71	9.63	0.16	0.016	0.840	0.935
Piglet mean BW, kg								
After cross-foster	1.29^ab^	1.20^a^	1.44^b^	1.41^b^	0.03	0.001	0.251	0.573
Day 7	2.45^b^	2.14^a^	2.80^c^	2.73^c^	0.06	0.000	0.053	0.209
Day 14	3.94^b^	3.51^a^	4.42^c^	4.30^bc^	0.09	0.000	0.074	0.315
Day 21	5.55^ab^	5.07^a^	6.16^b^	5.95^b^	0.11	0.001	0.102	0.506
Day 28	7.09^ab^	6.51^a^	7.69^b^	7.40^b^	0.13	0.003	0.079	0.539
Litter weight, kg								
After cross-foster	13.89	12.42	14.05	13.46	0.29	0.293	0.075	0.446
Day 7	25.50^b^	22.29^a^	26.94^b^	26.32^b^	0.56	0.011	0.073	0.219
Day 14	41.13^ab^	36.44^a^	42.82^b^	41.37^ab^	0.89	0.058	0.078	0.347
Day 21	57.81	52.50	59.51	57.46	1.22	0.172	0.131	0.502
Day 28	73.84	67.59	74.15	71.51	1.46	0.472	0.134	0.539
Mean litter weight gain, kg	59.95	55.16	60.09	58.05	1.33	0.575	0.208	0.611
Piglet mean ADG (g/day)								
Week 1	161.9^ab^	139.5^a^	192.4^b^	190.5^b^	6.10	0.001	0.278	0.361
Week 2	213.9	196.0	232.4	223.6	7.55	0.133	0.382	0.762
Week 3	229.6	222.0	247.5	235.8	8.54	0.367	0.584	0.908
Week 4	219.7	205.5	218.1	207.0	7.24	0.994	0.398	0.917
Overall	206.3^ab^	190.8^a^	222.6^b^	214.2^ab^	4.23	0.018	0.150	0.663
Average feed intake, kg	5.74^b^	5.54^ab^	5.43^ab^	5.28^a^	0.07	0.058	0.222	0.867

Sows were regarded as the experimental units, *n* = 18 for each group. Mean values with their standard error. Mean values within a row with unlike superscript letters were significantly different (*P* < 0.05). LE, low energy intake; HE, high energy intake; HAA, high amino acids intake; LAA, low amino acids intake; ADG, average daily gain.

### 3.4 Plasma biochemical profiles

**Figure 1:**
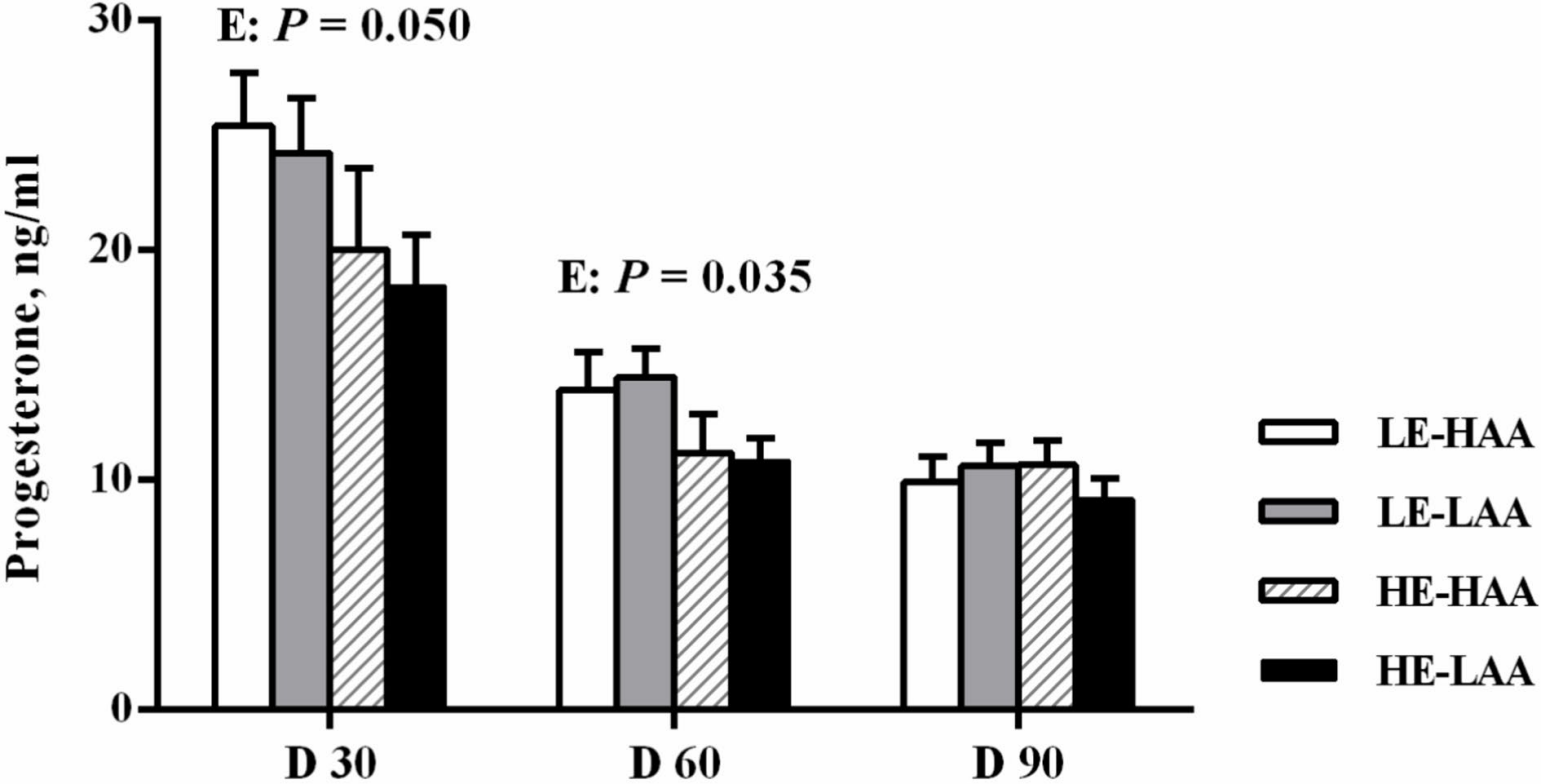
Effect of energy and amino acids intake during gestation on progesterone concentrations of sows. Mean values with their standard error. Mean values in a row with different letter are different (*p* < 0.05). LE, low energy intake; HE, high energy intake; LAA, low amino acids intake, HAA, high amino acids intake.

The plasma biochemical profiles are presented in Table 6. On day 110 of gestation, sows fed high energy had a higher plasma triglyceride content (*p* = 0.036), whereas sows fed high AA had a higher plasma Urea N concentration (*p* = 0.008). Sows fed high energy decreased the plasma progesterone concentrations on day 30 (*p* = 0.050; Figure 1) and 60 (*p* = 0.035; Figure 1) of gestation.

**Table 6:** Effects of energy and AA intake during gestation on blood biochemical profiles of sow.

Item	LE		HE		SEM	*p*-value		
	LAA	HAA	LAA	HAA		Energy	AA	Energy × AA
Breeding								
Urea N, mmol/L	2.01	2.23	2.34	2.21	0.11	0.492	0.839	0.455
Glucose, mmol/L	7.58	7.84	7.54	7.52	0.22	0.695	0.800	0.762
Triglyceride, mmol/L	1.33	1.39	1.31	1.33	0.11	0.861	0.852	0.942
Cholesterol, mmol/L	2.27	2.52	2.27	2.38	0.12	0.762	0.465	0.774
Day 110 of gestation								
Urea N, mmol/L	2.83^a^	3.43^b^	3.04^ab^	3.54^b^	0.10	0.415	0.008	0.810
Glucose, mmol/L	9.78	9.79	9.74	9.72	0.20	0.899	0.984	0.975
Triglyceride, mmol/L	1.49	1.53	1.73	1.80	0.06	0.036	0.594	0.901
Cholesterol, mmol/L	2.72	2.84	2.74	2.70	0.11	0.798	0.864	0.725
Weaning								
Urea N, mmol/L	3.45	3.67	3.78	3.65	0.14	0.492	0.839	0.455
Glucose, mmol/L	9.50	9.88	9.46	9.44	0.23	0.605	0.703	0.668
Triglyceride, mmol/L	1.73	1.69	1.54	1.52	0.10	0.401	0.880	0.969
Cholesterol, mmol/L	2.61	2.57	2.48	2.59	0.12	0.805	0.875	0.740

Sows were regarded as the experimental units, *n* = 8 for each group. Mean values with their standard error. Mean values within a row with unlike superscript letters were significantly different (*P* < 0.05). LE, low energy intake; HE, high energy intake; HAA, high amino acids intake; LAA, low amino acids intake.

### 3.5 Colostrum and milk composition

As shown in Table 7, the concentration of fat in colostrum was significantly higher (*p* = 0.001) in sows fed high energy than that in sows fed low energy. Moreover, sows fed high AA tended to increase (*p* = 0.068) the concentration of protein in the colostrum. There were no significant differences in the milk on day 14 and 28 of lactation among these four groups.

**Table 7:** Effect of energy and AA intake during gestation on colostrum and milk composition of sows.

Item	LE		HE		SEM	*p*-value		
	LAA	HAA	LAA	HAA		Energy	AA	Energy × AA
Colostrum								
Protein, %	16.63	17.59	16.70	18.03	0.31	0.684	0.068	0.769
Fat, %	4.24^a^	4.37^ab^	5.18^bc^	4.96^c^	0.12	0.001	0.831	0.440
Lactose, %	3.66	3.78	3.74	3.65	0.09	0.909	0.942	0.569
Solids-non-fat, %	21.21	22.32	21.39	22.64	0.37	0.739	0.120	0.925
Ash content, %	0.92	0.94	0.94	0.95	0.02	0.712	0.755	0.853
Total solid, %	25.46	26.69	26.57	27.60	0.42	0.235	0.186	0.900
Milk (L14)								
Protein, %	5.60	6.02	5.79	5.83	0.16	0.995	0.486	0.563
Fat, %	7.88	7.85	7.99	7.51	0.21	0.795	0.578	0.612
Lactose, %	5.03	5.16	5.04	4.96	0.19	0.804	0.952	0.788
Solids-non-fat, %	11.78	11.90	12.10	12.02	0.30	0.740	0.971	0.878
Ash content, %	0.95	1.06	1.06	1.02	0.03	0.502	0.439	0.176
Total solid, %	19.66	19.76	20.08	19.53	0.38	0.904	0.781	0.685
Milk (L28)								
Protein, %	4.88	4.77	4.82	5.13	0.06	0.236	0.432	0.101
Fat, %	7.38	7.45	7.20	7.01	0.16	0.356	0.854	0.689
Lactose, %	4.91	4.65	4.74	4.80	0.10	0.954	0.663	0.423
Solids-non-fat, %	10.75	10.31	10.51	10.92	0.13	0.489	0.961	0.123
Ash content, %	0.95	0.89	0.95	0.98	0.02	0.141	0.606	0.141
Total solid, %	18.13	17.86	17.71	17.93	0.20	0.774	0.863	0.500

Sows were regarded as the experimental units, *n* = 8 for each group. Mean values with their standard error. Mean values within a row with unlike superscript letters were significantly different (*P* < 0.05). LE, low energy intake; HE, high energy intake; HAA, high amino acids intake; LAA, low amino acids intake.

### 3.6 Redox status

Sows fed high energy decreased the concentrations of GPX and T-SOD on day 90 and the concentration of GPX on day 110 of gestation (*p* < 0.05; Table 8), and tended to increase (*p* = 0.057) the MDA on day 110 of gestation. Sows fed high AA had higher T-SOD concentrations on day 90 (*p* = 0.031) and 110 (*p* = 0.061) of gestation.

**Table 8:** Effect of energy and AA intake during gestation on plasma antioxidant indicators of sows.

Item	LE		HE		SEM	*p*-value		
	HAA	LAA	HAA	LAA		Energy	AA	Energy × AA
MDA, nmol/mL								
30 d	2.55	2.68	2.67	2.76	0.11	0.688	0.647	0.935
90 d	3.88	3.42	3.78	3.93	0.25	0.699	0.777	0.567
110 d	3.87	3.93	4.78	4.40	0.18	0.057	0.651	0.523
GPX, U/L								
30 d	914.9	884.5	926.0	895.0	16.7	0.761	0.392	0.993
90 d	803.5	806.4	748.1	735.9	15.5	0.048	0.878	0.803
110 d	713.1^c^	690.4^b,c^	621.6^a^	646.1^a,b^	12.3	0.004	0.967	0.270
T-SOD, U/mL								
30 d	37.45	37.18	37.66	36.70	0.30	0.825	0.336	0.584
90 d	33.11^c^	32.22^c^	29.25^b^	27.18^a^	0.57	<0.001	0.031	0.368
110 d	27.61	24.39	24.68	23.46	0.61	0.101	0.061	0.382

Sows were regarded as the experimental units, *n* = 8 for each group. Mean values with their standard error. Mean values within a row with unlike superscript letters were significantly different (*P* < 0.05). LE, low energy intake; HE, high energy intake; HAA, high amino acids intake; LAA, low amino acids intake. T-SOD, total superoxide dismutase; MDA, malondialdehyde; GPX, glutathione peroxidase.

### 3.7 Expressions of genes involved in nutrient transporter and oxidative stress in placenta

**Figure 2:**
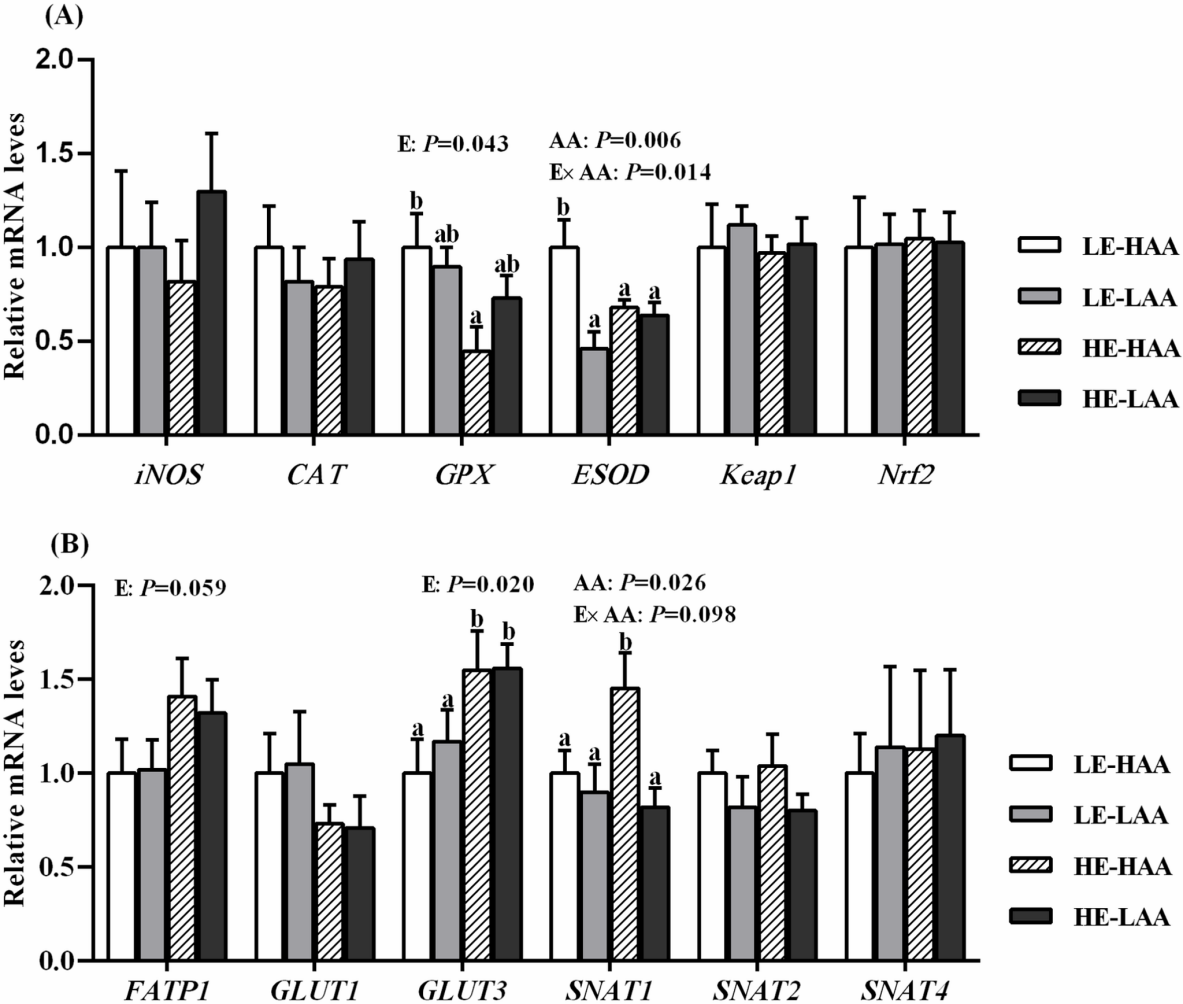
Effect of energy and AA intake during gestation on mRNA expressions of antioxidant-related genes **(A)** and nutrients transport-related genes **(B)** of sows. Mean values with their standard error. Mean values in a row with different letter are different (*p* < 0.05). Abbreviations: LE, low energy intake; HE, high energy intake; LAA, low amino acids intake, HAA, high amino acids intake; CAT, catalase; iNOS, nitric oxide synthase 2, inducible; GPX, glutathione peroxidase; ESOD, extracellular superoxide dismutase; Nrf2, nuclear erythroid 2-related factor 2; Keap1, Kelch-like ECH-associated protein 1; GLUT, glucose transporter; FATP, fatty acids transport protein; SNAT, sodium-dependent neutral amino acid transporter.

Sows fed high energy decrease the mRNA abundance of GPX (*p* = 0.043; Figure 2A) but increased the mRNA abundance of FATP1 (*p* = 0.059; Figure 2B) and GLUT3 (*p* = 0.020; Figure 2B). Moreover, sows fed high AA increased the mRNA abundance of ESOD (*p* = 0.006; Figure 2A) and SNAT2 (*p* = 0.026; Figure 2B). In addition, the Energy × AA interaction affected the mRNA abundance of ESOD (*p* = 0.014; Figure 2A) and SNAT1 (*p* = 0.098; Figure 2B).

### 3.8 Body change during lactation

Sows fed high energy or high AA intake during gestation decreased lactational BW loss, respectively (*p* < 0.01; Table 9). Consistently, the calculated lactational fat and protein loss decreased by the enhanced high energy or high AA intake during gestation (*p* < 0.01).

**Table 9:** Sow live weight, backfat, and calculated protein and fat mass during lactation.

Item	LE		HE		SEM	*p*-value		
	HAA	LAA	HAA	LAA		Energy	AA	Energy × AA
Farrowing (Kg)	263.57	251.64	269.23	263.90	3.29	0.177	0.193	0.617
Weaning (Kg)	249.64	245.29	238.64	240.87	2.68	0.159	0.844	0.544
Lactation BW loss (Kg)	13.76^b^	6.35^a^	30.55^d^	23.01^c^	1.55	<0.001	<0.001	0.972
Lactation BW loss (%)	5.01^b^	2.48^a^	11.33^d^	8.56^c^	0.55	<0.001	<0.001	0.855
Farrowing (mm)	20.86	20.43	21.71	20.93	0.34	0.332	0.388	0.801
Weaning (mm)	19.71	19.64	18.86	19.33	0.31	0.363	0.752	0.669
Lactation loss (mm)	1.16^ab^	0.74^a^	2.75^c^	1.63^b^	0.12	<0.001	<0.001	0.041
At parturition								
Fat mass (Kg)	57.35	54.20	59.60	57.33	1.09	0.222	0.219	0.841
Protein mass (Kg)	40.36	38.47	41.06	40.43	0.48	0.169	0.195	0.514
At weaning								
Fat mass (Kg)	52.89	51.87	49.46	50.28	0.92	0.182	0.957	0.621
Protein mass (Kg)	38.39	37.63	36.75	37.05	0.39	0.164	0.769	0.509
Calculated sow fat mass								
Lactation fat loss (Kg)	4.46^b^	2.33^a^	10.13^d^	7.05^c^	0.48	<0.001	<0.001	0.426
Lactation fat loss (%)	7.27^b^	4.36^a^	17.09^d^	12.06^c^	0.78	<0.001	<0.001	0.256
Calculated sow protein mass								
Lactation protein loss (Kg)	1.97^b^	0.84^a^	4.31^d^	3.39^c^	0.23	<0.001	0.001	0.727
Lactation protein loss (%)	4.70^b^	2.11^a^	10.47^d^	8.23^c^	0.53	<0.001	0.001	0.789

Sows were regarded as the experimental units, *n* = 18 for each group. Mean values with their standard error. Mean values within a row with unlike superscript letters were significantly different (*P* < 0.05). LE, low energy intake; HE, high energy intake; HAA, high amino acids intake; LAA, low amino acids intake.

### 3.9 Correlations

**Figure 3:**
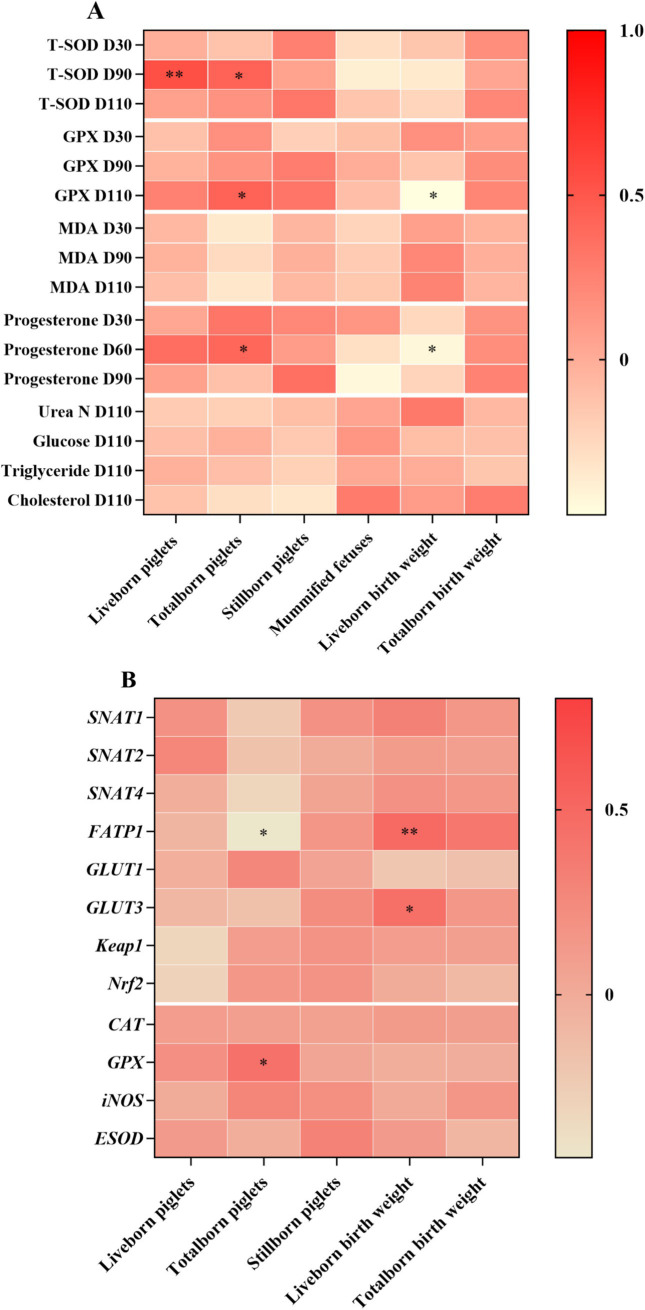
Heatmaps of correlation of reproductive performance and blood parameters **(A)** and gene expressions in the placenta **(B)** of sows. T-SOD, total superoxide dismutase; MDA, malondialdehyde; GPX, glutathione peroxidase; CAT, catalase; iNOS, nitric oxide synthase 2, inducible; ESOD, extracellular superoxide dismutase; Nrf2, nuclear erythroid 2-related factor 2; Keap1, Kelch-like ECH-associated protein 1; GLUT, glucose transporter; FATP, fatty acids transport protein; SNAT, sodium-dependent neutral amino acid transporter. *p < 0.05; **p < 0.01.

Correlation analysis was performed to evaluate the potential link between reproductive performance and blood parameters and gene expressions in the placenta (Figure 3). Plasma T-SOD at day 90 of gestation was positively correlated with live born piglets and total born piglets. Moreover, plasma GPX at day 110 of gestation and plasma progesterone at day 60 of gestation were positively correlated with total born piglets, whereas they were negatively correlated with live born piglets birth weight (Figure 3A). Live born piglets birth weight was positively correlated with the mRNA abundance of FATP1 and GULT3. Total born piglets was negatively correlated with the mRNA abundance of FATP1, whereas it was positively correlated with the mRNA abundance of GPX (Figure 3B).

## 4 Discussion

Litter size is primarily influenced by the survival rate of embryos during early gestation (21). In this study, sows fed high energy during gestation exhibited a lower number of live born piglets and an increased occurrence of mummified fetus. These findings align with previous studies indicating that a high feeding level or increased energy intake during early gestation is associated with reduced embryo survival (22, 23). High energy intake in early gestation has been shown to diminish systemic progesterone concentrations by elevating the progesterone metabolic clearance rate, which adversely affects endometrial secretory functions and leads to increased embryo mortality (24). In line with this, sows with high energy intake demonstrated decreased concentrations of progesterone at days 30 and 60 of gestation in the current study.

In the current study, high AA and energy intake markedly enhanced plasma urea N and triglyceride at day 110 of gestation, respectively. The increased metabolic burdens on sows during late gestation elevated systemic oxidative stress (25). Notably, an imbalance between antioxidants and the production of reactive oxygen species (ROS) is considered responsible for fetal survival during gestation and reduces reproductive performance (26). In this study, sows with high energy intake exhibited lower plasma concentrations of GPX and T-SOD and higher plasma concentrations of MDA compared to sows with low energy intake during late gestation. Consistent with this, our previous study demonstrated that high energy intake during late gestation resulted in increased MDA accumulation in sows (6). In contrast, sows with high AA intake showed an increase in plasma T-SOD concentration during late gestation. As primary antioxidants, GPX and T-SOD serve as the first line of defense against ROS (17). MDA is a toxic lipid metabolite produced as a consequence of ROS (27), and the elevated MDA levels in sows with high energy intake indicate increased lipid peroxidation. Maternal effects on the fetus are first mediated by the placenta, with these effects then observed in the fetus. Accordingly, the mRNA expressions of antioxidant related in the placenta were determined, and we found that sows with high energy intake exhibited decreased mRNA expression of GPX in the placenta. These results suggested that high energy intake during gestation may exacerbate oxidative stress in late gestation, whereas increased amino acid intake may enhance the body’s antioxidant capacity.

Consistent with previous studies (10, 28), increased energy intake during gestation resulted in higher birth weights of both total born and live born piglets. Along with the higher birth weight, piglets form sows fed high energy had higher organ weights compared to those form sows fed low energy. Interestingly, the relative weight of brain was lighter in piglets form sows fed high energy than those form sows fed low energy. This phenotypic change could be further explained by selectively allocating maternal nutrition to optimize the growth of internal organs (29, 30). Fetal growth in utero depends primarily on the placenta to transport maternal nutrients to the fetus. The proper development and function of placenta are crucial for the growth and survival of the developing fetus in utero (31). To delve further into this matter, we measured the mRNA expressions of nutrient transporter in the placenta. We found that sows with high energy intake during gestation exhibited higher mRNA expressions of FATP1 and GLUT3 in the placenta compared to those with low energy intake, while high AA intake increased the mRNA expression of SNAT1 in the placenta. In addition, both high energy and AA intake had a interaction on the mRNA expression of SNAT1. Glucose and amino acids serve as substrates for both fetal growth and placental development (31). In the placenta, GLUT3 is responsible for glucose transport, while SNAT1 primarily facilitates the uptake of neutral amino acids (32, 33). Placenta FATP1 is one of the major fatty acid transporters responsible for facilitating and regulating cellular fatty acid uptake (34). Altogether, our study demonstrated that increased birth weight may be partly attributed to the upregulation of key genes related to nutrient transporter induced by changes in energy or AA intake.

The composition of sow colostrum was closely related to the nutritional intake and body condition of sows during late gestation (35). Previous studies have indicated that increased lysine intake is associated with higher protein concentrations in the colostrum of sows (2, 36). Similarly, the fat content in colostrum has been shown to rise with elevated dietary energy level during gestation (37). In the present study, high AA or high energy intake during gestation consistently resulted in increased protein and fat concentrations in the colostrum, respectively. These findings align with our previous study, which demonstrated that enhanced nutritional levels during gestation led to increase both protein and fat content of colostrum in sows (38). Consequently, the increased colostrum composition supports the higher ADG of piglets from sows with high energy intake during the first week of lactation. Additionally, piglets from sows fed high AA exhibited greater weight compared to those from sows fed low AA on day 28 of lactation. The feed intake of sows during lactation is a significant factor affecting milk composition (39). However, we observed no effect on milk composition at day 14 and day 28 of lactation among the four treatment groups, even though the ADFI in HE-LAA sows was significantly lower than LE-HAA sows. We speculate that a substantial fraction of fat from sow is deposited into milk, as evidenced by the greater lactational body weight loss in HE-LAA sows. Piglets with higher birth weight exhibited stronger suckling competition compared to their littermates, contributing to higher weaning weight (40). In this study, piglets form sows with high energy intake had higher birth weight and presented larger overall ADG. However, due to variations in initial litter size, the average litter weight gain did not differ among the four treatment groups.

In this study, increased energy and amino acid intake during gestation significantly increased body weight gain and back fat thickness in high-parity sows. A positive relationship has been established between body weight gain during gestation and body weight loss during lactation (8). Accordingly, we observed that lactational body weight loss was significantly exacerbated by increased energy or AA intake during gestation, with the HE-HAA group exhibiting the most pronounced weight loss due to the cumulative effects of elevated energy and AA intake. This excessive body weight loss during lactation could potentially lead to abnormal fetal development, litter non-uniformity and variation in piglet birth weight in the next litter (13, 41). Therefore, overestimating high energy and AA intake during gestation may be detrimental to reproductive performance in the subsequent parity.

## 5 Conclusion

In this study, high-parity sows (between parities 7 and 9) fed with high energy intake during gestation decreased live born piglets and increased piglets birth weight, which likely was associated with the change of peripheral progesterone, antioxidant ability and placental nutrient transport. Moreover, sows with increased intake of AA during gestation lead to better redox status and litter performance, which may be attributed to improvements in antioxidant capacity and colostrum composition.

## Data Availability

The datasets presented in this study can be found in online repositories. The names of the repository/repositories and accession number(s) can be found in the article/supplementary material.
